# The Dental Protective Association of the United States

**Published:** 1889-10

**Authors:** 


### EDITORIAL.

THE Dental Protective Association of the United States was organized for the purpose of ascertaining and maintaining the rights and interest of the dental profession against demands made under patents, the validity of which is in dispute and have not been fully established.

The right to do this can not be justly disputed by any. As to what may rightfully be done in carrying out such a purpose, there is hardly room for difference of opinion; it certainly is not the best course to contribute to the support of the claimants who act under such questionable patents. It is often the case that persons by acts of acquiescence place themselves under tribute to those who hold invalid patents without inquiry, simply from fear of being involved in litigation, or at least unenviable notoriety.

The claims of the International Tooth-Crown Co. are being pressed with energy, while the validity of their patents is still in question.

It would seem that the best, and proper thing for this Co. to do, if they have faith in the validity of their patents, is to press a test case to a final issue in the courts, and in this way settle the matter, once for all; that would end all contest, and the affairs of the Co. would then proceed in a harmonious and business-like manner.

From the present aspect of the matter it is quite certain that the dentists of the country will not rest satisfied short of a definite settlement of the questions involved, and the sooner that is done the better for all parties concerned. The temper of the profession in respect to this matter is pretty clearly indicated by action had at the recent meeting of the American Dental Association, held at Saratoga. From its proceedings we give the following:

"In view of the injustice which the profession has sustained in the past, as well as the annoyances to which it may be subjected in the future, and fully appreciating the arduous and important labors of Dr. J. N. Crouse, of Chicago, be it

"*Resolved*, That Dr. Crouse is entitled to the earnest and practical support of every dentist in the United States, and further be it

"*Resolved*, That the American Dental Association fully approves of the formation, the plans and methods of the Dental Protective Association of the United States, and pledge to it, our united and continued support and moral aid."

It is to be hoped that every dentist in the country who has the interest of his profession at heart, will stand by, and cooperate with, his professional brethren, in accordance with the above resolutions.

